# The Legal Strength of International Health Instruments - What It Brings to Global Health Governance?

**DOI:** 10.15171/ijhpm.2016.122

**Published:** 2016-09-04

**Authors:** Haik Nikogosian, Ilona Kickbusch

**Affiliations:** ^1^World Health Organization Regional Office for Europe, Copenhagen, Denmark.; ^2^Global Health Centre, Graduate Institute for International and Development Studies, Geneva, Switzerland.

**Keywords:** International Health Instruments, Legal, Treaties, Impact, Health Governance

## Abstract

Public health instruments have been under constant development and renewal for decades. International legal instruments, with their binding character and strength, have a special place in this development. The start of the 21st century saw, in particular, the birth of the first World Health Organization (WHO)-era health treaties – the WHO Framework Convention on Tobacco Control (WHO FCTC) and its first Protocol. The authors analyze the potential impact of these instruments on global health governance and public health, beyond the traditional view of their impact on tobacco control. Overall, the very fact that globally binding treaties in modern-era health were feasible has accelerated the debate and expectations for an expanded role of international legal regimes in public health. The impact of treaties has also been notable in global health architecture as the novel instruments required novel institutions to govern their implementation. The legal power of the WHO FCTC has enabled rapid adoption of further instruments to promote its implementation, thus, enhancing the international instrumentarium for health, and it has also prompted stronger role for national legislation on health. Notably, the Convention has elevated several traditionally challenging public health features to the level of international legal obligations. It has also revealed how the legal power of the international health instrument can be utilized in safeguarding the interests of health in the face of competing agendas and legal disputes at both the domestic and international levels. Lastly, the legal power of health instruments is associated with their potential impact not only on health but also beyond; the recently adopted Protocol to Eliminate Illicit Trade in Tobacco Products may best exemplify this matter. The first treaty experiences of the 21st century may provide important lessons for the role of legal instruments in addressing the unfolding challenges in global health.

Public health instruments have been under constant development and renewal for decades, to address the increasing complexity of determinants and drivers of health. Globalization and the growing impact of transnational factors on health have accelerated the demand for new types of international instruments aimed at expanded strength, coverage, and compliance.

International legal instruments for health, with their binding character and strength, have a special place in the above development. The start of the 21st century saw, in particular, the birth of the first World Health Organization (WHO)-era health treaties – the WHO Framework Convention on Tobacco Control (WHO FCTC)^1^ and its first Protocol (the Protocol to Eliminate Illicit Trade in Tobacco Products)^2^ - to supplement the international instrumentarium for health. The impact of this process on global health governance is relatively new but nonetheless wide-ranging and significant.

Indeed, even as the WHO FCTC and its Protocol were negotiated and adopted with the principal objective of strengthening the global action against tobacco, it can also be argued that they opened a new phase in WHO-era global health that accepted international legally binding treaties as one major way forward and that they constituted a breakthrough by revealing new types of processes, institutions and instruments, as described below.

While there has been a significant literature that maintains that some other major developments, such as the international response to HIV/AIDS, changed global health profoundly, the impact of the first treaties on how we “see and do” global health is still largely unexplored. Nonetheless, some observations on what the first WHO-era treaties bring to global health governance and broader public health, beyond their impact on tobacco control, can already be made. This article aims at summarizing the authors’ preliminary observations and conclusions, based on a review of treaty measures and developments that have the potential for overarching public health impact, recognizing also that further research in the coming years would bring more details and depth to the topic.

First, the negotiations and adoption of the FCTC, WHO’s first international convention, “unlocked” the treaty- making power of WHO contained in its Constitution^3^ but never previously used. The Convention, in turn, promptly demonstrated its own treaty-making power by giving birth to its first Protocol, a new international treaty in its own right. Overall, the very fact that globally binding instruments in health were feasible has accelerated the debate and expectations for an expanded role of international legal regimes in public health.

Second, the impact of treaties has been notable in global health architecture. The novel instruments required novel institutions to govern their implementation. The Conference of the Parties (COP) is the Convention’s central organ and governing body, comprised of all Parties. In addition, Parties established the Convention Secretariat, the permanent executive arm, which functions within WHO but is directly accountable to the COP on treaty matters. The recently adopted Protocol, which is a new international treaty in its own right, will have its own governing body, the Meeting of the Parties (MOP) once it enters into force. These are new types of international bodies in public health which both enrich and influence the global health architecture and governance.

Third, the impact on enhancing the system of international public health instruments has been notable. The legal power of the Convention has enabled rapid adoption of further instruments to promote its implementation, such as the guidelines on most substantive articles,^4^ the reporting system, and most recently the first Protocol. What is also peculiar is that all these instruments have resulted from formal intergovernmental processes established by the treaty’s governing body – an important feature underlining their technical and political strengths alike.

Fourth, the WHO FCTC prompted a stronger role for national legislation for health**.** Indeed, international law can in general be effectively implemented when translated into domestic law. In the case of the FCTC, 80% of Parties either adopted new tobacco control legislation or strengthened their existing laws after ratifying the Convention.^5^ In a noteworthy development, some Parties utilized the treaty’s legal power to enact legal acts even in areas (surveillance, public education, etc) that were previously regulated through “softer” means (such as national guidelines and various administrative acts). These processes, although resulting from a specific international instrument, do also promote more understanding of and role for legal acts in public health.

As another feature directly linked to health governance, the WHO FCTC has elevated several important public health features to the level of international legal obligations, particularly those concerning reporting, national coordination mechanism, international cooperation, and protecting public policies from industry interference. These are key but generally challenging functions in public health. The strengthening of these functions in relation to tobacco control would, therefore, inspire their bolstering in broader public health.

For example, the binding obligation with regard to regular reports on implementation of the Convention resulted in a stable global implementation review and monitoring system, which is an important but not always easy task in public health. The legal obligation to establish a national coordination mechanism for tobacco control is inspiring similar mechanisms in broader public health frameworks, particularly for health promotion and the prevention and control of non-communicable diseases (NCDs) in many countries. The obligation to cooperate bilaterally and through multilateral platforms is resulting in enhanced mechanisms for implementation assistance as well as in development of human capital and diplomacy in negotiating and promoting health. Lastly, the obligation to act against tobacco industry interference has demonstrated how a treaty provision would not only bind but also empower governments in protecting their public policies.

The impact of treaties and treaty-making in health have also been notable in at least three other areas:

The impact on multisectoral cooperation and the whole-of-government approach to health deserves a special mention. Apart from empowering the national coordination mechanism, already mentioned above, the WHO FCTC, and importantly also its Protocol, have substantially expanded the spectrum of different sectors involved in negotiations and action for public health. The empowerment that treaties bring to multisectoral collaboration for health is, thus, multifaceted and substantial, as also are the gains that the health sector and health ministries will acquire in such interdisciplinary engagement for public health.

The WHO FCTC has also revealed how the legal power of an international health instrument can be utilized in safeguarding the interests of health in the face of competing agendas and legal disputes, at both the domestic and international levels. It has been applied by governments when responding to tobacco industry claims and threats, and in deciding to proceed with tobacco control measures notwithstanding these claims and threats, citing its obligation under or the power it gets from the FCTC, and it has also been applied by courts when upholding national tobacco control acts legally challenged by the tobacco industry.^6,7^

Lastly, the legal power of health instruments is associated with their potential impact not only on health but also beyond. The recently adopted Protocol to Eliminate Illicit Trade in Tobacco Products may best exemplify this matter. Illicit trade not only fuels the tobacco epidemic but also causes substantial losses to governments’ revenues. It also creates serious challenges to customs operations and supply chain control, and to crime prevention and the fight against organized crime in general; not only is the tobacco illicit trade in most cases run by organized criminal groups, but the illegal profits obtained from it are used to fund other criminal activities in countries and internationally. Therefore, while the future implementation of the Protocol would contribute to public health as its main objective, it would also contribute to areas such as customs, revenue collection and economies and to crime prevention and security in general.

International agreements, commitments and partnerships are an integral part of every nation’s global health engagement.^8^ Overall, there should be little doubt that the international health instrumentarium of the 21st century will further expand and develop, with binding and “soft” instruments alike, to address the changing landscape of public health. This would further enrich the diplomacy and governance for global health. Health treaties have their unique role in this process, as the experiences of the two first treaties of the new century have shown. These are the experiences to keep in mind while the new century, with its own challenges to public health, unfolds.

## Ethical issues

Not applicable.

## Competing interests

The authors declare that they have no competing interests.

## Authors’ contributions

HN is the former (2007-2014) Head of the Secretariat of the WHO Framework Convention on Tobacco Control. He has prepared the core draft of the article, which was then discussed and finalized jointly with IK whose particular contribution was the viewpoint from global health diplomacy and governance. The authors also regularly collaborate under the umbrella of the Global Health Centre of the Graduate Institute for International and Development Studies, Geneva, Switzerland led by IK.

## Authors’ affiliations

^1^World Health Organization Regional Office for Europe, Copenhagen, Denmark. ^2^Global Health Centre, Graduate Institute for International and Development Studies, Geneva, Switzerland.

## References

[1] WHO Framework Convention on Tobacco Control Geneva: World Health Organization; 2003. WHO website. http://www.who.int/fctc/text_download/en/. Accessed July 6, 2016.

[2] WHO Framework Convention on Tobacco Control. Protocol to Eliminate Illicit Trade in Tobacco Products. Geneva: World Health Organization; 2013. WHO website. http://apps.who.int/iris/bitstream/10665/80873/1/9789241505246_eng.pdf?ua=1. Accessed July 6, 2016.

[3] Constitution of the World Health Organization. WHO website. http://apps.who.int/gb/bd/PDF/bd48/basic-documents-48th-edition-en.pdf. Accessed July 6, 2016.

[4] Guidelines for implementation of the WHO Framework Convention on Tobacco Control. WHO website. http://www.who.int/fctc/treaty_instruments/adopted/guidel_2011/en/. Accessed July 6, 2016.

[5] 2014 Global progress report on implementation of the WHO Framework Convention on Tobacco Control. WHO website. http://apps.who.int/gb/fctc/PDF/cop6/FCTC_COP6_5-en.pdf. Accessed July 6, 2016.

[6] The High Court of Justice decision on UK standardized packaging: Key points for other jurisdictions. McCabe Centre for Law and Cancer web site. http://www.mccabecentre.org/blog/uk-decision.html. Accessed July 6, 2016.

[7] Judiciary of England and Wales: British American Tobacco and others – v- Department of Health. Summary of judgement by Mr Justice Green; Queen’s Bench Division of the High Court, Administrative Court. https://www.judiciary.gov.uk/wp-content/uploads/2016/05/bat-v-doh-summary.pdf. Accessed August 23, 2016. Published May 19, 2016.

[8] Kates J, Katz R (2011). The role of treaties, agreements, conventions, and other international instruments in global health. Infect Dis Clin N Am.

